# Examining the Dose–Response Relationship between Outdoor Jogging and Physical Health of Youths: A Long-Term Experimental Study in Campus Green Space

**DOI:** 10.3390/ijerph19095648

**Published:** 2022-05-06

**Authors:** Yuheng Mao, Yichen He, Tianyu Xia, Haorun Xu, Shuai Zhou, Jinguang Zhang

**Affiliations:** 1 The College of Landscape Architecture, Nanjing Forestry University, Nanjing 210037, China; maoyuheng@njfu.edu.cn (Y.M.); xty0710@njfu.edu.cn (T.X.); 2 Faculty of Science, National University of Singapore, Singapore 119077, Singapore; e0732876@u.nus.edu; 3 College of Civil Engineering, Nanjing Forestry University, Nanjing 210037, China; xuhaorun@njfu.edu.cn; 4 Asia-Europe Institute, University of Malaya, Kuala Lumpur 50603, Malaysia; s2040929@siswa.um.edu.my

**Keywords:** campus green space, outdoor physical activity, physical health, activity tracker, physical activity intervention, natural experiment

## Abstract

Many studies have demonstrated that outdoor physical activity positively affects the physical health of young people. Here, we aimed to examine the extent to which outdoor jogging was associated with the physical health of youths, and then to decipher whether a dose–response relationship exists between them. A total of 2852 youths from a Chinese university were enrolled in a long-term experimental study between September 2018 and September 2019. We conducted two waves of physical health tests for 2852 youths (before and after the jogging interventions in 2018 and 2019, respectively) using China’s National Student Physical Health Standard (NSPHS). Paired *t*-tests were used to examine statistical differences. A multiple regression model was used to evaluate the associations between jogging and physical health. The results showed that: statistically significant changes in the two waves of physical health outcomes were suggested after jogging interventions; outdoor jogging in campus green space was associated with participants’ physical health after controlling for covariates; and a dose–response relationship between jogging and physical health outcomes was revealed, with 120–140 km/year (approximately 3.43–4 km/week) being the most effective intervention dose. Our findings have implications for promoting physical health in youth groups by encouraging outdoor physical activity.

## 1. Introduction

### 1.1. Physical Activity and Health Benefits

The rapid pace of urban life, the increasing pressure of social competition, screen-based media use, and sedentary lifestyles have led to a lack of physical activity, leading to various health problems [1,2]. Many studies have examined the association between physical activity and various health outcomes [3]. For example, a cross-sectional study of adolescents in the United States and Canada suggested a significant relationship between physical activity and various positive health indicators, such as health status, self-image, quality of life, and interpersonal relationships [4]. With growing scientific evidence on the health benefits of physical activity [5,6], many physical activity intervention experiments have emerged [7]. In previous studies, the research participants included adolescents, children, older adults, and patients with specific diseases, whereas outcome variables included mental health, well-being, and physical health [8,9,10,11]. Physical activity intervention research for youth mainly focuses on psychological perspectives, such as self-cognition, emotion, and pressure [12,13,14]. However, associations between physical activity interventions and physical health responses targeting the youth groups have seldom been mentioned. 

### 1.2. Physical Activity Scenarios Comparison

Physical activity scenarios can be divided into indoor and outdoor scenarios. Scholars have emphasised the importance of the outdoor environment in promoting physical activity and health benefits [15]. In a comparative study of the health-promoting effects of indoor and outdoor physical activity, scholars found that outdoor physical activity in a natural setting had better effects on physical and mental health [16]. Moreover, previous studies demonstrated that outdoor physical activities were better for restoring attention and physical activity engagement, yielding lower levels of physical exhaustion and eliciting higher positive and lower negative effects compared with indoor physical activities [17,18,19]. Epidemiological studies have also confirmed that outdoor physical activity can reduce the risk of diseases associated with specific populations [20], and enhance mental well-being and quality of life in different age groups [21,22,23,24]. 

### 1.3. Physical Activity in Green Spaces

Green space is a vital component of the outdoor environment, and includes greenways, parks, campus green spaces, and other urban green infrastructure. An increasing number of scholars have focused on people’s behaviour, psychological status, physical health, and other aspects of benefits in green space exposure [25,26,27]. For example, a series of green intervention studies by a research team from Wuhan, China, confirmed significant changes in health indicators such as walking behaviour, physical activity, and the mental status of residents before and after a surrounding urban park was built [28,29,30]. Similarly, cross-sectional research in Turkey reported that adolescents’ green exercise (GE) and health could be promoted with urban green spaces (UGSs) [31]. As green space matters to people in various ways, we cannot ignore the influence of green space when conducting physical activity intervention and daily physical activity behaviour research [32].

Previous studies related to green spaces and physical activity have mainly focused on physical activity intention and the amount of physical activity [33]. For instance, Schipperijn et al. used a regression model to examine the relationship between green space exposure and residents’ physical activity behaviour in Denmark, proving a certain relationship between them [34]. An Australian study of middle-aged and older adults showed that the amount of green space could improve people’s physical activity behaviour [35]. In a Florida study, researchers found that the amount of green space within a given distance, and the total amount of green space in an area, can boost residents’ athletic behaviour [36]. A team from Shanghai, China, found that the green view index (GVI) increases the likelihood of physical activity [37]. However, most of the studies in this field used convenient cross-sectional designs, and very few studies conducted the natural experiments from a long-term follow-up perspective.

Additionally, researchers have discussed the health benefits of green space [38] without focusing on the physical health benefits from physical activity in green space, and have studied how green space promotes physical activity [39] without focusing on the link between physical activity and physical health in green space. In short, there is an ignored gap in the existing literature: physical activity intervention experiments in green spaces are scarce, not to mention dosing studies in this area, which is not conducive to the formulation of school physical activity policies.

In addition to the lack of physical activity intervention experiments in green spaces, most studies on green space physical activity have focused on residents, such as older adults and teenagers. We observed very little research on youths. Some studies have found that the physical activity situation of youths is worrying, resulting in overweight, obesity, and other harm to physical health; thus, this is worthy of our attention [40]. Previous studies conducted in schools among teenagers and children have shown that physical activity interventions in schools positively affect physical health [41]. However, there are challenges to implementing school policies [42,43,44].

Most studies on the classification of green spaces have focused on green spaces in parks, streets, and communities. Studies of campus green spaces are rare. In the existing studies on youths and campus green spaces, most perspectives focus on mental health, life pressure, and academic performance, using statistical methods such as the mediation model, regression model, and space syntax [45,46,47,48,49,50,51,52]. However, there are still gaps among studies on the relationship between outdoor physical activity and physical health in green campus spaces.

### 1.4. Our Study

To fill the gaps mentioned above, we conducted a 1-year natural experiment (2018–2019) at the Nanjing Forestry University, China, where campus green space coverage is relatively high. A natural experimental study design, sometimes called the pre-post-intervention design, is often used to evaluate the benefits of specific interventions [53]. Researchers and policymakers regard natural experiments as a superior approach to evaluating the health impact of interventions compared with cross-sectional research designs. Notably, natural experiments are different from full experimental methods such as randomised controlled trials (RCTs). In RCTs, where the exposed and non-exposed groups are identified, participants are randomly assigned to a differentiated group under the control of the researchers. Experiments are based on things that happen in natural phenomena. In public health research, interventions are difficult to manipulate in many situations. Using a natural experiment method, we compared the physical health levels before and after the physical activity intervention. The researchers could still control all participants in the natural experiment: the exposed (jogging normally) and unexposed groups (jogging not much). Admittedly, natural experiments have biases and limited internal validity compared with RCTs. However, they have better external validity, and the assignment of participants seems to be random, better reflecting real-life situations.

This experiment contributes to the study of outdoor physical activity and health in green spaces. In particular, this is a natural experiment in universities in developing countries, and almost all natural experimental studies have been conducted in developed countries. Through this study, we can explore the influence of contemporary Chinese youths’ outdoor physical activity in green spaces on physical health to provide guidance on urban green space development for government leaders and researchers, help policymakers better formulate relevant physical activity guidance policies, and provide college students with suggestions in the field of physical activity in the future.

In this experiment, 2852 participants (first- and second-year students) experienced outdoor jogging in campus green spaces using activity trackers. The participants were divided into nine groups according to treatment variables to examine the dose–response relationship between outdoor jogging and physical health. A natural experiment was conducted to address the following questions:

- Is long-term outdoor jogging in campus green spaces positively associated with youths’ physical health?
- What dose of outdoor jogging in campus green spaces can achieve the best health-promoting effects?

## 2. Materials and Methods

### 2.1. Study Area

Our experimental area was the Xinzhuang Campus of the Nanjing Forestry University, located in Xuanwu District, Nanjing, covering an area of 0.838 km^2^. The campus has a long history of being rich in plant species, and has a high green coverage rate. There were 384 plant species belonging to 93 families and 218 genera. The school district is near Purple Mountain and Xuanwu Lake, a beautiful environment surrounded by mountains. The wonderful site condition is in line with the requirements of campus green space, which is suitable for carrying out natural experiments.

The city of Nanjing (118°46′ E, 32°03′ N) is one of the metropolitans in China, with an administrative area of 6598 km^2^, of which 46.50% is covered by vegetation [54]. Nanjing has many colleges and universities. By 2021, there will be 53 undergraduate colleges and universities in Nanjing, with 918,100 college students ranking high in China. University research in Nanjing has high representativeness and academic value.

**Figure 1:**
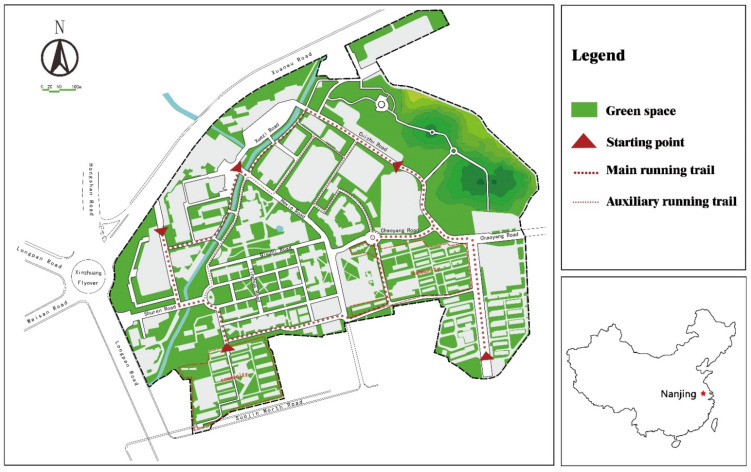
Study area with the trail network.

The wonderful site condition(Figure 1) and representative samples provide good conditions for our upcoming natural experiments.

### 2.2. Sampling Design

To evaluate the impact of outdoor physical activity in green space on physical health, we conducted two waves of physical health tests on participants, in autumn 2018 and autumn 2019, according to China’s National Student Physical Health Standard (NSPHS). Participants’ physical health was tested before and after the intervention.

This study used a natural experimental pre- and post-test design conducted between September 2018 and January 2019. Pre- and post-test designs are one of the most common methods for testing the effectiveness of an intervention. The physical activity intervention lasted from 2018 to 2019. Nanjing has a humid subtropical climate heavily influenced by the East Asia Monsoon, leading to hot, rainy summers, and cold, dry winters. Therefore, Nanjing’s autumn climate and temperature conditions were suitable for physical health tests. In addition, we put the test time on the day when there was no rain, reasonably evaluating the participants’ physical health. As a result, the 2852 participants performed exceptionally well on both physical health tests.

We used China’s NSPHS as the criterion for the two test waves. This standard was formulated by the Ministry of Education of the People’s Republic of China to evaluate students’ physical health monitoring. The criteria were selected to reflect the basic state of body composition, cardiovascular function, muscle strength, muscle endurance, and joint and muscle flexibility, which are closely related to physical health. The test measures body shape, body function, and physical fitness, such as height, weight, body mass index (BMI), lung capacity, 50-m run, sitting forward, and long-distance running. In view of the different evaluation indices for different age groups, we adopted the evaluation indices for college students, and calculated their total score according to the China’s NSPHS [55].

Participants performed outdoor jogging for up to 1 year and wore activity trackers to record physical activity behaviour. We used the Budao fun running software to track the movement. Based on the global position system (GPS) and sensor technology, the software accurately records the track, mileage, pace, step number, and other movement data, intuitively displaying the entire process of students’ movement. Furthermore, the software adopts the running mode of orienteering, which enhances the fun of sports and effectively prevents cheating. In addition, the software was combined with artificial intelligence technology to automatically analyse the background track, step number, and other data to effectively judge cheating behaviour.

To illustrate the dose–response of different outdoor jogging mileages in campus green spaces among youths, we divided 2852 youths into nine groups (below 100 km, 100–120 km, 120–140 km, 140–160 km, 160–180 km, 180–200 km, 200–220 km, 220–240 km, and over 240 km). The grouping followed the maximum possible isometric selection interval to ensure scientific grouping. The dose to physical activity was defined as a continuous measure based on the total outdoor jogging mileage. A continuous measurement can accurately assess the dose level to the physical activity, compared with categorical measurement, simply defining the exposed and unexposed in terms of total outdoor jogging mileage.

We rigorously followed 2852 youths by conducting two waves of physical health tests. To make the experiment more objective, we used the first wave of physical health records as the initial physical health. In addition, we recorded the age, sex, and other indicators of the participants.

### 2.3. Data

The first wave of China’s NSPHS test score can be used to describe participants’ initial physical health level. In the second wave, according to China’s NSPHS test score as the outcome variable, after the year-long physical activity intervention in campus green space, their physical health level became the outcome variable index. We tracked the participants’ outdoor physical activity behaviour for 1 year by detecting and tracking outdoor jogging behaviour. Total outdoor jogging mileage of participants was taken as a treatment variable. We took the first wave health outcome indicator, sex, and age as individual-level covariates in the model. As described, the fitting degree and objectivity of the model can be enhanced. Subsequently, data analysis and screening were performed for grouping and dose–response studies.

### 2.4. Statistical Analyses

First, correlation analyses were conducted on the total outdoor jogging mileage, duration, and frequency of the three statistical indicators. Before further analysis, for scientific experimentation, we compared the differences between the two waves of China’s NSPHS test scores using the paired *t*-test. The participants’ two waves of China’s NSPHS test scores and individual factors in different groups were reported using means and standard deviations (SDs) for continuous variables, and percentages for categorical variables. Notably, Statistical Package for the Social Science (SPSS) 26.0 was used for the correlation analysis and paired *t*-test.

We employed multivariate regression using the ordinary least squares model. The multivariate regression model (1) can explain physical health after the physical activity intervention experiment. After our initial attempt, the model was highly fit; therefore, the study was conducted. Multicollinearity among covariates was assessed using variance inflation factors (VIFs). Statistical regression analyses were performed using STATA 16.0.

(1) $$\begin{array}{cc} Physical\;health_{ic} & \\ & =\beta_{0}+\beta_{1}mileage_{ic}+\beta_{2}sex_{ic}+\beta_{3}age_{ic}\\ & +\beta_{4}Initial\;physical\;health_{ic}+\varepsilon_{ic} \end{array}$$

$Physical\;health_{ic}$ represents the second wave of China’s NSPHS test score of participant *i* in group *c*; $\beta_{1}$ describes the influence coefficient of outdoor jogging in campus green spaces on physical health; $\beta_{2}$ describes the influence coefficient of sex on physical health; we use dummy variables, setting males to 1 and females to 0; $\beta_{3}$ describes the influence coefficient of age on physical health; $\beta_{4}$ describes the influence coefficient of initial physical health before physical activity intervention on physical health; $\varepsilon_{ic}$ is the individual-level error term. In Model (1), if $mileage_{ic}$ is statistically significant (*p* < 0.05), it can be concluded that the outdoor jogging intervention has a significant impact on the participants’ physical health.

We divided 2852 students into nine groups and substituted them into a Model (1) to observe the changes in *β*_1_ and explore the changes in dose–response.

Suppose that the model settings in different sample groups are the same. In this case, the coefficient sizes between the two groups can be compared, which is necessary for most empirical analyses [56]. However, we cannot be sure that the difference is statistically significant. This is because their 95% confidence intervals (CIs) overlapped. Interaction terms (Chow test) were introduced to test the coefficient difference between the groups. Therefore, dummy variables and interaction terms were introduced to conduct an inter-group test of the coefficient difference. The regression equation is shown in Equation (2):

(2) $$\begin{array}{cc} Physical\;health_{ic} & \\ & =\varphi +\overset{9}{\underset{c=2}{\sum }}\alpha_{c}group_{ic}+\delta_{c}mileage_{ic}\\ & +\overset{9}{\underset{c=2}{\sum }}\gamma_{c}group_{ic}\times mileage_{ic}+\sigma_{ic}individual_{ic}+\varepsilon_{ic} \end{array}$$

$Physical\;health_{ic}$ represents the second wave of China’s NSPHS test scores for participant *i* in group *c*. $\alpha_{c}$ represents the fixed effect of the group. $group_{ic}$ represents the second wave of China’s NSPHS test scores for participant *i* in group *c*. $\alpha_{c}$ represents the fixed effect of the group. $group_{ic}$ represents the dummy variable for student *i* in group *c*. The base group was set as the Group 1, and the other groups were arranged in order. Mileage represents student *i*’s total outdoor jogging mileage. $\gamma_{c}$ represents the interaction coefficient before the total mileage of outdoor jogging in the campus green spaces and grouping. We used the significance of this index (*p* < 0.05) to determine the differences between the groups. If it is significant, the coefficient size relationship in Model (1) is valid. Because the significant groups were tested for coefficient sizes, we selected two baseline groups, and reported significance. $\sigma_{ic}$ is the coefficient of the individual control covariates. $\varepsilon_{ic}$ is the individual-level error term.

Here, we report the significance of the interaction terms. Based on the significance of the interaction terms, we can determine whether the coefficient size relationship is valid.

## 3. Results

### 3.1. Correlation Analysis

Correlation analyses were conducted on the total outdoor jogging mileage, duration, and frequency of the three statistical indicators. The Pearson correlation coefficient between total outdoor jogging mileage and duration among our participants was 0.924. The Pearson correlation coefficient between the total outdoor jogging duration and frequency was 0.885. The Pearson correlation coefficient between the total outdoor jogging mileage and frequency was 0.904. We can observe that the three indicators are significantly correlated. Therefore, we chose one treatment variable for the three highly correlated variables. As for the description of the amount of physical activity, our long-term observation of students showed that some students stopped at the same place, but kept timing. The total outdoor jogging duration was not objective, and the outdoor jogging frequency could not fully express students’ physical activity amount and intensity. This correlation analysis demonstrates that selecting the total outdoor jogging mileage for the dose–response analysis is reasonable.

### 3.2. Significant Change of the Two Waves of Tests

The descriptive statistics are presented in Table 1. Paired *t*-tests indicated significant differences in the changes in the two waves of the national student physical health standard test scores (*p* < 0.05). We can observe that the changes in the two waves of tests were significant for the overall groups. Except for the <100 km group, all the other groups showed positive changes. Most of the selected personnel were first- and second-year students at the individual level, and their average age was between 18 and 19 years. Women made up a relatively large proportion of the total sample (62.2%). We can see that the highest mileage was concentrated in a higher percentage of male youths.

### 3.3. Regression Estimates

The VIFs values were <2, indicating no multicollinearity issues. Table 2 summarises the regression results. If the outdoor jogging mileage variable is statistically significant, it suggests that outdoor jogging in campus green spaces greatly affects the participants’ physical health. In the overall sample population, the effect of outdoor physical activity was significant. Specifically, the group results (with a total mileage of 100–120 km, 120–140 km, 140–160 km, and 160–180 km) are still significant, whereas the other groups show insignificant results. $\beta_{1}$ describes the influence coefficient of the influence of outdoor jogging in campus green spaces on physical health. In the groups of significance, the influence coefficient shows a changing trend, first increasing and then decreasing, reaching a peak in the group with a total mileage of 120–140 km (0.101). The initial physical health test score was not significant in the group with a total mileage below 100 km, but significant in the other groups. In the group with a total mileage of over 240 km, the influence coefficient of the initial physical health test score was very large, indicating that in the group of students with excellent physical activity habits, outdoor jogging in green spaces for 1 year had little impact on their physical health. This could even have a slightly negative effect.

### 3.4. Chow Test

**Figure 2:**
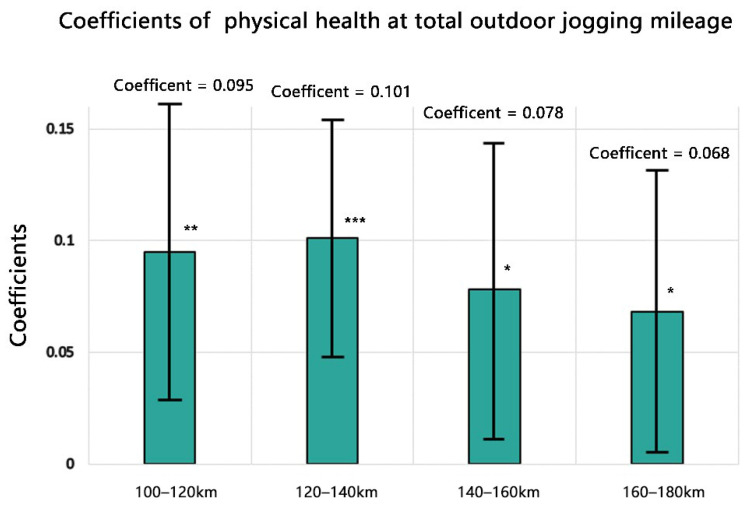
Histograms of the coefficients of total outdoor jogging mileage on physical health. * *p* < 0.1, ** *p* < 0.05, *** *p* < 0.01.

As can be seen from Table 3, the results of the interaction terms are all significant, indicating that a relationship between the coefficient sizes is established. This result indicates that the coefficient size relationship is valid and verifies the robustness of our analysis. A histogram of the coefficient size relationship is shown in Figure 2, and 95% confidence intervals (CIs) are reported.

## 4. Discussion

In this research, we conducted a natural experiment to explore the dose–response relationship between jogging and physical health in campus green space in Nanjing, China. Moreover, the most effective intervention dose was revealed.

Though the positive effect of jogging on the health of the population has been demonstrated in previous studies many times, the specific space where the physical activity takes place should be emphasised, especially in the green spaces of a modern city.

### 4.1. The Physical Health Change Results from Physical Activity Intervention

Our natural experiment at the Nanjing Forestry University showed that outdoor physical activity in campus green spaces positively affected physical health. At the end of the year-long physical activity intervention, the data showed an average increase in physical health score of 1.75, which was significant. Our study results are consistent with those of a large number of physical activity interventions. However, these studies were conducted in older adults, children, and people with specific diseases or jobs [57,58,59,60]. However, some specific exercise interventions, such as high-intensity interval training (HIIT) [61] based on school-based activities, show insignificant weight and other physical health indices. These differences are attributed to sampling people and physical activity types [62]. Physical activity intervention experiments vary widely among samples and populations, but are generally effective [63].

Our study has significant implications for physical activity in green spaces. We conducted year-long observations and two waves of physical health tests on a vegetated campus. Many participants and testers entered our experiment, a large-scale natural experiment. However, many physical activity intervention studies have adopted RCTs [64,65]. The sample size of RCTs is often small, and further discussion and exploration are needed to validate the results. Under the conditions of physical activity intervention, it is of great significance to adopt natural experimental methods that reflect the real world. A large natural experiment on policy-based physical activity intervention in Brazil concluded that physical activity intervention could enhance physical activity time. Leisure-time physical activity (LTPA) was assessed using the international physical activity questionnaire (IPAQ). However, the effects of exercise interventions have not been studied in a natural experiment [66]. Our experimental study demonstrated a positive effect on physical health.

In addition, for the selection of explanatory variables, most studies focus on single indicators such as obesity [67]. We tried to break through and make a more comprehensive evaluation with the help of various indicators of China’s NSPHS. In this case, we found a significant increase in the youth group, showing that year-long outdoor jogging in green spaces is feasible and worth advocating.

### 4.2. Dose–Response Relationship

We conducted a rare study on physical health and physical activity dose–response in green space physical activity. Instead of simply dividing the exposed and non-exposed groups, we observed the physical activity dose of the participants by dividing the groups and conducting studies. We determined a threshold for the impact of green space outdoor jogging behaviour on physical health. Previous studies have usually not considered the existence of a threshold. Most experiments were designed as linear regression models for large-scale intervention experiments. The total number of samples was substituted into the study; thus, drawing a monotonous conclusion, indicating negative, positive, significant, or insignificant. However, this is generally problematic. Natural experiments suggest the possibility of performing dose–response grouping studies. Outdoor jogging in green space had the greatest impact on physical health among students whose outdoor jogging total mileage in green space was 120–140 km. According to the number of experimental weeks, 3 km was the best jogging distance per week for most people. Although the result is not significant for the group whose running mileage is less than 100 km, we can infer through the coefficient that the influence is very small. This is consistent with previous studies on the variable dose–response relationships between exercise training and performance. An inverted U-shaped relationship was demonstrated between the exercise effect and the amount of exercise [68].

Because the mileage of the natural experiment was voluntary, we should also focus on the group with relatively high mileage. We believe that this group had superior athletic and physical activity habits. We found that the physical activity intervention presented a different situation for the groups with excellent physical activity habits. With the increase in outdoor jogging mileage, we found that the influence coefficient of the total mileage over the 240 km group was negative, but insignificant. In contrast, the initial physical health of the participants in this group had a significant influence on the results. Therefore, it can be inferred that participants in this group had good physical activity habits, and outdoor jogging in green spaces had less impact on their physical health than other physical activities.

In contrast, the jogging intervention in green spaces did not significantly increase the physical health of the group with excellent physical activity habits. Therefore, for those with excellent exercise habits and physical health, to maintain physical health, more scientific and systematic exercise is needed; otherwise, there will be a slight regression. Furthermore, different types of physical activity interventions have different effects on participants with high physical activity [69]. Jogging is not a good choice. Simultaneously, we should be aware of the negative effects of overtraining, and look for moderate amounts of exercise [70].

### 4.3. Strength and Limitations

This study has several advantages. First, we tracked the physical activity situation using electronic devices, avoiding deviation from previous questionnaires. The respondents’ expressions and self-evaluations were subjective [71,72]. As research on activity trackers has emerged in recent years, we can choose a more accurate method [73]; the data are objective and scientific. The motion tracking system is based on portable accelerometers and a GPS. Second, based on the results of the investigators, we present the exact distribution. We further conducted a grouping study to explore the response of the physical activity dose to physical health. What is more advantageous is that our two waves of physical health testers were all professional Chinese NSPHS testers with rich testing experience; thus, their experimental results were highly reliable.

We must also acknowledge that there are many limitations to our study. First, we considered personal-based control variables, but could not monitor physical activity other than outdoor jogging. We firmly believe that activities other than outdoor jogging, such as basketball, swimming, and indoor fitness activities, still significantly impact participants’ physical health in our natural experiment. These factors should be included in the multiple regression model as covariates. However, we were unable to observe this aspect of physical activity. Second, our sample consisted of a large number of students from one school. The campus is relatively single; thus, it is impossible to conduct a systematic study on the various indicators of green space. We will try to conduct a detailed study considering different environments in multiple schools and campuses. Third, the study was 1 year; longer tests may yield more objective and long-term results. Finally, we cannot avoid factual errors in the experiment, such as the presence of infectious diseases in the test participants, which can affect the test results. Chronic diseases that affect physical health and behaviour cannot be monitored.

### 4.4. Future Directions

Based on our findings, we provide feasible suggestions for future research. Dose–response research on green space physical activity intervention is still very scarce at present. We often enter a general study on a certain kind of physical activity behaviour to draw the following conclusion: negative or positive. Most studies in this field have been cross-sectional [74], and a questionnaire survey is a universal evaluation method. However, experiments based on activity tracks are an inevitable trend in this field. Based on the study of physical activity dose–response on physical health in green spaces, sample size and type need to be expanded, which is also the focus of this study. Simultaneously, more systematic construction of environmental indicators and variables should be included. Moreover, the research group should extend the green foundation design of urban campuses, and focus on more diverse urban green infrastructure such as parks. Green space physical activity intervention is a topic that needs continuous attention and research in academic circles, and has a profound impact on urban planning [75] and the health-related policies of residents.

## 5. Conclusions

Our robust study (conducted at the Xinzhuang Campus of Nanjing Forestry University), based on a grouping regression model, showed that outdoor jogging in green spaces has a positive effect on physical health, and the effect had a threshold, peaking at 120–140 km/year (3.43–4 km/week).

Our dose study adopted an experimental study design, based on activity trackers, establishing robust scientific causality evidence between green spaces and physical activity. This is of symbolic significance for the construction of a green campus infrastructure and student movement policies in the future.

In summary, our findings highlight the feasibility and possibility of green space outdoor physical activity for physical health, and bridge the gaps in previous studies. Policy guidance can be based on our research to improve youths’ urban life, physical health, and all residents through urban planning.

## Figures and Tables

**Table 1:** Descriptive statistics of participants by total outdoor jogging mileage (*N* = 2852).

	Baseline Mean (SD)/%	Follow-up Mean (SD)/%	Change Mean (SD)/%	Age Mean (SD)/%	Sex (%female)	*N*
Below 100 km	77.19 (8.82)	76.20 (9.60)	−0.99 (4.36) **	18.49 (0.82)	67.1%	170
100–120 km	76.67 (5.85)	77.23 (6.05)	0.56 (3.57) **	18.31 (0.69)	85.5%	427
120–140 km	75.53 (6.72)	76.92 (6.39)	1.39 (4.07) ***	18.38 (0.75)	58.6%	669
140–160 km	74.78 (7.50)	77.21 (6.80)	2.43 (4.61) ***	18.41 (0.72)	58.1%	456
160–180 km	75.57 (6.80)	76.84 (6.58)	1.27 (4.05) ***	18.25 (0.60)	65.2%	451
180-200 km	74.00 (7.89)	77.34 (7.01)	3.34 (4.74) ***	18.31 (0.76)	58.0%	331
200–220 km	74.24 (7.73)	77.73 (7.09)	3.49 (4.16) ***	18.29 (0.74)	50.0%	192
220–240 km	72.86 (8.10)	76.62 (7.46)	3.76 (4.25) ***	18.40 (0.70)	41.7%	96
Over 240 km	75.00 (6.96)	77.85 (7.04)	2.85 (3.25) ***	18.55 (0.75)	28.3%	60
Overall	75.32 (7.21)	77.07 (6.84)	1.75 (4.35) ***	18.35 (0.72)	62.2%	2852

Note: ** *p* < 0.01, *** *p* < 0.001.

**Table 2:** Regression estimates of outdoor physical activity in green space on physical health.

	Mileage Beta (95% CI)	Sex Beta (95% CI)	Age Beta (95% CI)	Initial Physical Health Beta (95% CI)	Constant	R^2^	*N*
Below 100 km	0.008 (−0.010, 0.026)	0.616 (−0.894, 2.126)	−0.440 (−1.342, 0.463)	0.961 (0.899, 1.024) ***	9.506	0.798	170
100–120 km	0.095 (0.029, 0.160) **	−1.289 (−2.373, −0.206) *	0.100 (−0.435, 0.636)	0.837 (0.770, 0.905) ***	0.870	0.683	427
120–140 km	0.101 (0.048, 0.154) ***	−0.158 (−0.795, 0.479)	0.141 (−0.220, 0.501)	0.768 (0.715, 0.821) ***	3.312	0.662	669
140–160 km	0.078 (0.012, 0.144) *	−0.979 (−1.889, −0.070) *	−0.021 (−0.542, 0.500)	0.687 (0.626, 0.748) ***	15.049	0.643	456
160–180 km	0.067 (0.006, 0.131) *	−0.306 (−1.303, 0.690)	1.106 (0.506, 1.705) ***	0.784 (0.698, 0.870) ***	−14.135	0.682	451
180–200 km	0.052 (−0.019, 0.123)	−0.680 (−1.741, 0.392)	0.282 (−0.306, 0.871)	0.700 (0.619, 0.781) ***	10.823	0.652	331
200–220 km	0.050 (−0.043, 0.144)	0.577 (−0.632, 1.787)	0.289 (−0.417, 0.995)	0.784 (0.693, 0.876) ***	3.396	0.720	192
220–240 km	0.050 (−0.091, 0.191)	0.354 (−1.411, 2.119)	0.080 (−1.104, 1.263)	0.795 (0.681, 0.908) ***	5.660	0.731	96
Over 240 km	−0.010 (−0.026, 0.006)	1.420 (−0.489, 3.329)	-0.933 (−1.927, 0.060)	0.960 (0.836, 1.084) ***	24.813	0.813	60
Overall	0.020 (0.017, 0.024) ***	−0.194 (−0.546, 0.158)	0.167 (−0.039, 0.374)	0.777 (0.749, 0.805) ***	12.500	0.673	2852

Note: * *p* < 0.05, ** *p* < 0.01, *** *p* < 0.001.

**Table 3:** Chow test.

	*p*-Value with Group 3 as Reference	*p*-Value with Group 4 as Reference
Group 2 * mileage	0.044 *	0.042 *
Group 3 * mileage		0.000 ***
Group 4 * mileage	0.047 *	
Group 5 * mileage	0.034 *	0.034 *

Note: * *p* < 0.05, *** *p* < 0.001.

## Data Availability

The data presented in this study are available upon request from the corresponding author. The data are not publicly available due to ethical and legal restrictions imposed by the Institutional Review Board of Department of Physical Education, Nanjing Forestry University.
